# The Association of Sleep Duration and Quality with Heart Rate Variability and Blood Pressure

**Published:** 2020-11

**Authors:** Amirreza Sajjadieh, Ali Shahsavari, Ali Safaei, Thomas Penzel, Christoph Schoebel, Ingo Fietze, Nafiseh Mozafarian, Babak Amra, Roya Kelishadi

**Affiliations:** 1 Department of Internal Medicine, Isfahan University of Medical Sciences, Isfahan, Iran,; 2 Medical Student, Isfahan University of Medical Sciences, Isfahan, Iran,; 3 Center of Sleep Medicine, Charite e Universitatsmedizin€ Berlin, Berlin, Germany,; 4 Charite e Universitatsmedizin€ Berlin, Department of Cardiology and Pulmonology, Center of Sleep Medicine, Berlin, Germany,; 5 Child Growth and Development Research Center, Research Institute for Primordial Prevention of Non-Communicable Disease, Isfahan University of Medical Sciences, Isfahan, Iran,; 6 Department of Pulmonology, Bamdad Research Center, Isfahan University of Medical Sciences, Isfahan, Iran

**Keywords:** Sleep duration, Sleep quality, Heart rate variability, Blood pressure

## Abstract

**Background:**

The current study was conducted to evaluate the relation of sleep duration and quality with blood pressure (BP) and heart rate variability (HRV).

**Materials and Methods:**

This cross-sectional study was carried out in 2017 among 260 staff of a university hospital in Isfahan, Iran. They were selected by multi-stage random method from different wards. Time domain spectral analysis was used to measure a number of HRV parameters. The long-term components of the HRV were estimated using the standard deviation of the normal-to-normal interval (SDNN). The square root of the mean squared differences of successive NN intervals (RMSSD) was calculated by statistical time domain measurements; SNN50, and PNN50 were measured. Pittsburg sleep quality index (PSQI) questionnaire was used to assess sleep quality.

**Results:**

Higher PSQI score correlated with lower SDANN rise (OR=0.92). Fairly bad to very good subjective sleep quality had association with lower SDANN (OR=0.43). Very high sleep latency to very low sleep latency ratio had association with lower SDANN (OR=0.39) and lower PNN50 (OR= 0.44). Sleep duration and HRV parameters had no significant association. Fairly bad sleep efficiency to very good sleep efficiency ratio was correlated with lower SDANN (OR= 0.29). Very high daytime dysfunction to very low daytime dysfunction ratio had correlation with lower SDANN (OR=0.35). Very bad compared to very good subjective sleep quality had significant correlation with higher Heart rate (HR) (B=0.03). Very high sleep latency compared to no sleep latency was associated with higher HR (B=4.74). Very high compared to very low amount of sleep disturbances correlated with higher SBP levels (B=15.2). Using sleep medication less than once a week compared with no history of taking such drugs was associated with higher HR (B=16.4).

**Conclusion:**

Our findings showed that poor sleep quality are adversely associated with HRV, HR and BP. This finding should be considered in clinical and preventive recommendations.

## INTRODUCTION

Sleep, as a physiological resting state, is seriously in correlation with human being health as its process needs a variety of molecular and organ biological regulation and integrity (1, 2). This mental and physical resting state is required for healthy functioning of human being including behavioral, developmental and intellectual functioning (1, 3, 4).

Mammals including human beings spend at least one third of their lifetime sleeping. This fact may strongly reflect the fundamental process of sleeping and its effects on different body organs, while long ago it was considered that sleeping is only a body quite state (2, 4).

Variety of medullar, cortical and subcortical neural network cooperation as well as hormonal and co-ordinated neurotransmission secretion are needed to maintain sleep quality. In addition to the mentioned factors, the Autonomic nervous system (ANS) plays a key role during sleep process and is responsible for cardiovascular function variability during sleeping including its onset and stage transitions (4, 5).

Beat-to-beat modulation of Heart rate (HR) occurs via ANS activity by Sympathetic nervous system (SNS) and Parasympathetic nervous system (PNS) discharges through sympathetic and vagal innervations of the heart, respectively (6, 7).

Heart rate variability (HRV) during sleeping is a great matter of debate. Although it is known that HR would decrease during sleep, this overall pattern is influenced with body circadian temperature, stage of sleep, body movements and awakenings. It is confirmed that during non-rapid eye movement stage (NREM) of sleep, parasympathetic cardiac innervations is more active and cardiovascular system is stable, while during REM stage, cardiovascular system is steeply affected by sympathetic system (8, 9).

Studies have confirmed that sleep deprivation can affect persons' cardiovascular system functions negatively and also may be associated with hypertension in future in both adults and children (6, 10, 11). In addition, reduced HRV has been shown to be in association with cardiovascular diseases. Moreover, it has been confirmed that HRV abnormality is independently a mortality risk factor (7, 12).

The goal of this study is to evaluate the association of sleep duration and quality with Blood pressure (BP) and HRV.

## MATERIALS AND METHODS

The current cross-sectional study was performed among the staff of Alzahra Hospital in Isfahan, Iran. Sampling was done in a multi-stage random method based on the different wards of the hospital. Total population of the study was 260. All participants were asked about medication use and diseases. Participants with a history of major medical and psychiatric disorder or drug consumption were omitted from the study. Prior to the HRV measurements, the individuals under study were asked to answer a questionnaire on their demographic data and life habits (e.g., smoking, coffee drinking, and exercise). The measurement tool was Arshia Crown Teb (ACT) and software was Cardiorhythm A.C.T V3.7b.

Data analysis was performed using STATA (version 11.0, STATA Corp, College Station, TX, USA). Informed consent was given by all participants.

### Data gathering methods:

#### Blood pressure: (13)

BP measurement was done while participants were calm and in a sitting position for 30 minutes. BP was measured twice with a standardized and calibrated mercury sphygmomanometer from the right arm. The interval between two measurements was five minutes. An average of two measurements for systolic and diastolic BP were recorded.

Hypertension was diagnosed in participants based on the 90th percentile of the distribution of SBP and/or DBP with accordance to gender, age, and height.

### Heart rate variability:

The subjects were prohibited from alcohol and caffeine consumption after 10 p.m. (22:00) on the night before the HRV measurements. In addition, smokers were asked to avoid smoking in the hour before the measurements. To control diurnal variation, HRV measurement was performed between 8 a.m. (08:00) and 12 a.m. (12:00). Electrodes were placed on each subject’s wrists and left foot to derive HRV for 5 min, while sitting comfortably on a chair and breathing ordinarily.

The HRV parameters measured by time domain spectral analysis included the following: The standard deviation of the normal-to-normal interval (SDNN) used to estimate long-term components of the HRV, and the square root of the mean squared differences of successive NN intervals (RMSSD) acquired by statistical time domain measurements. SNN50, and PNN50 were measured among the participants.

### Assessment of sleep quality:

Assessment of sleep quality was done among participants using the Pittsburg sleep quality index (PSQI) questionnaire. Each participant was instructed to fill in questions regarding their respective subjective sleep quality, sleep duration, sleep latency, habitual sleep efficiency, use of sleeping medication, sleep disturbances and daytime dysfunction as the 7 components of the Pittsburg index.

## RESULTS

Of all 260 participants, 114 (43.8%) were males. Mean age (SD) was 38.5(13.3) years and mean BMI was 25(4.6) Kg/m^2^. Mean sleep duration among participants was 6.1(1.7) hours a day. 105 of the participants had poor quality sleep. Mean heart rate was 85.1 in females and 79.2 in males. Systolic and diastolic BP was not significantly different among males and females. Mean (SD) PSQI global score was 5.9(3.5) and 7.3(3.7) in males and females, respectively (Table 1).

**Table 1.** Demographic and clinical characteristics of participants

**Characteristics**	**Total Mean(SD)**	**Male**	**Female**	**P-Value**
**Age, Mean (SD)**	38.57(13.3)	35 (13.1)	41.3(12.7)	<0.001
**Body mass index (kg/m^2^ )**	25(4.6)	23.9(3.8)	25.8(4.9)	<0.001
**Systolic blood pressure (mmHg)**	123.59(16.20)	124.2(13.98)	122.8(17.5)	0.50
**Diastolic blood pressure (mmHg)**	75.45(12.16)	76(11.5)	75.00(12.68)	0.52
**Average heart rate (beats/min)**	82.45(11.32)	79.24(12.4)	85.1(9.6)	<0.001
**Self-reported sleep duration (h/night)**	6.1(1.74)	6.3(1.67)	5.8(1.76)	0.02
**Time of going to bed (hr PM)**	9.12(4.04)	9.07(3.92)	9.15(4.15)	0.88
**Time of getting up (hr AM)**	6.74(1.78)	6.76(1.86)	6.73(1.73)	0.90
**PSQI global score**	6.6(3.7)	5.9(3.5)	7.3(3.7)	0.01
**PSQI**				
**GOOD <5h (0) N(%)**	78(42.6)	40(50)	38(36.9)	0.08^‡^
**POOR >5h (1) N(%)**	105(40.4)	40(50)	65(63.1)	
**Time domain**				
**SDANN(ms)**	39.2(33.2)	44.3(39.1)	34.9(26.7)	0.13^*^
**RMSSD(ms)**	20.6(17.7)	21.9(20.4)	19.7(15.5)	0.69^*^
**SNN50(ms)**	12.7(23.4)	16.9(28.2)	9.5(18.3)	0.16^*^
**PNN50(%)**	5.2(9.3)	7.3(11.7)	3.5(6.4)	0.12^*^

* According to the Mann-Whitney test,

‡ According to the chi-square test, other p-values are based on t-test

PSQI: Pittsburgh Sleep Quality Index, SD: standard deviation, Sys: Systolic blood pressure, Dia: diastolic blood pressure, Mean heart rate (MHR)(1/min)

The tertiles of SDANN, RMSSD, SNN50 and PNN50 are presented in Table 2. As presented in Table 3, higher PSQI score was associated with lowering the chance of SDANN rise (OR=0.92). Fairly bad to very good subjective sleep quality ratio was associated with lower SDANN amount (OR=0.43). Very high sleep latency to very low sleep latency ratio was significantly associated with lower SDANN amount (OR=0.39) and also with lower PNN50 amount (OR= 0.44). A significant relationship between sleep duration and HRV parameters was not found. Fairly bad sleep efficiency to very good sleep efficiency ratio was also correlated with lower SDANN amount (OR= 0.29). Very high daytime dysfunction to very low daytime dysfunction ratio was significantly associated with lower SDANN amount (OR=0.35). Linear regression predicting SBP, DBP and MHR parameters by sleep data showed that very bad compared to very good subjective sleep quality correlated significantly with higher HR among participants (B=0.03). Moreover, very high sleep latency was compared to no sleep latency, correlating with higher HR (B=4.74). In the crude model sleep duration of less than 5 hours compared to more than 7 hours was associated with higher SBP levels (B=7.3); even though this relationship was not significant after adjusting for age, sex and BMI. Very high compared to very low amount of sleep disturbances was associated with higher SBP levels in participants (B=15.2). As presented in Table 4, using sleep medication (less than once a week) as compared with no history of taking such drugs was associated with higher HR (B=16.4).

**Table 2.** Tertiles of heart rate variability

**Time domain**	**Tertile 1**	**Tertile 2**	**Tertile 3**
**SDANN(ms)**	<=20	21–51	>51
**RMSSD(ms)**	<=12	13–25	>=26
**SNN50(ms)**	0	1–9	>=10
**PNN50(%)**	0	0.2–3.60	>=3.60

SDANN: SD of the 5-minute average N-N intervals; RMSSD: Square root of the mean of the squares of successive N-N interval differences; SNN50 The number of N-N intervals >50 ms different from preceding interval; PNN50: The percentage of intervals >50 ms different from the preceding interval

**Table 3.** Ordinal regression predicting heart rate variability parameters by sleep data

	**SDANN**				**RMSSD**				**SNN50**				**PNN50**			

	OR^a^	p-Value^a^	OR^b^	p-value^b^	OR^a^	p-Value^a^	OR^b^	p-Value^b^	OR^a^	p-Value^a^	OR^b^	p-Value^b^	OR^a^	p-Value^a^	OR^b^	p-Value^b^
**PSQI(continuous)**	0.92	0.02	0.92	0.03	0.95	0.22	0.96	0.35	0.95	0.16	0.96	0.28	0.93	0.06	0.95	0.19
**PSQI**																
**Good(N= 80)<5 0**	1	0.009	1	0.008	1	0.19	1	0.22	1	0.07	1	0.09	1	0.04	1	0.10
**Poor(N=104) >5 1**	0.48		0.46		0.69		0.70		0.60		0.62		0.57		0.62	
**Subjective sleep quality**																
**New-E9**																
**Very good (0)**	1		1		1		1		1		1		1		1	
**Fairly good (1)**	0.64	0.13	0.62	0.11	0.86	0.61	0.78	0.42	1.10	0.74	1.05	0.87	0.91	0.74	0.90	0.74
**Fairly bad (2)**	0.50	0.049	0.43	0.02	0.55	0.11	0.51	0.08	0.67	0.27	0.67	0.28	0.57	0.13	0.59	0.17
**Very bad(3)**	0.78	0.62	0.77	0.63	2.11	0.16	2.04	0.19	1.40	0.51	1.49	0.44	1.57	0.39	1.79	0.28
**Sleep latency**																
**Very low(0)**	1		1		1		1		1		1		1		1	
**Fairly low(1)**	0.70	0.23	0.69	0.22	0.62	0.11	0.64	0.13	0.62	0.11	0.65	0.15	0.66	0.16	0.69	0.21
**Fairly high(2)**	0.53	0.06	0.48	0.04	0.66	0.23	0.65	0.23	0.86	0.65	0.89	0.73	0.74	0.36	0.73	0.35
**Very high(3)**	0.41	0.03	0.39	0.02	0.46	0.07	0.50	0.10	0.41	0.03	0.46	0.06	0.40	0.03	0.44	0.05
**Sleep duration**																
**>=7 h (0)**	1		1		1		1		1		1		1		1	
**6–7 h (1)**	1.19	0.65	1.15	0.72	1.28	0.53	1.19	0.67	1.11	0.79	1.04	0.92	1.08	0.84	1.07	0.87
**5–6 h (2)**	0.74	0.36	0.76	0.41	2.29	0.42	1.26	0.49	1.21	0.54	1.17	0.63	1.37	0.32	1.46	0.25
**<5 h (3)**	0.58	0.14	0.64	0.26	0.8	0.55	0.83	0.64	0.65	0.24	0.68	0.32	0.68	0.29	0.79	0.55
**Habitual sleep efficiency**																
**>85% (0)**	1		1		1		1		1		1		1		1	
**75– 84% (1)**	0.64	0.21	0.79	0.52	0.99	0.97	1.04	0.93	0.91	0.80	0.97	0.94	0.78	0.47	0.87	0.71
**65–74% (2)**	0.28	0.006	0.29	0.007	0.58	0.20	0.57	0.19	0.63	0.28	0.63	0.29	0.67	0.35	0.71	0.43
**<65% (3)**	0.90	0.76	0.98	0.95	0.54	0.09	0.57	0.13	0.52	0.06	0.56	0.11	0.53	0.07	0.60	0.16
**Sleep disturbances**																
**Very low(0)**	1		1		1		1		1		1		1		1	
**Fairly low(1)**	0.85	0.75	0.84	0.76	0.52	0.23	0.45	0.16	0.68	0.46	0.62	0.38	0.58	0.31	0.52	0.25
**Fairly high(2)**	0.52	0.25	0.44	0.16	0.61	0.41	0.51	0.28	0.63	0.41	0.58	0.36	0.52	0.26	0.48	0.23
**Very High(3)**	0.58	0.47	0.58	0.50	0.46	0.33	0.55	0.49	0.47	0.32	0.62	0.55	0.39	0.23	0.53	0.44
**Use of sleeping medication**																
**Not during the past month (0)**	1		1		1		1		1		1		1		1	
**Less than once a week(1)**	1.01	0.99	0.88	0.86	1.08	0.92	1.06	0.93	0.83	0.77	0.82	0.75	1.04	0.95	1.04	0.95
**Once or twice a week(2)**	2.75	0.26	2.65	0.29	1.65	0.54	1.64	0.54	2.91	0.24	3.53	0.18	1.58	0.57	1.94	0.43
**Three or more times a week(3)**	0.90	0.81	1.13	0.79	0.82	0.67	0.84	0.71	0.85	0.71	0.89	0.79	0.67	0.35	0.72	0.47
**Daytime dysfunction**																
**Very low(0)**	1		1		1		1		1		1		1		1	
**Fairly low(1)**	0.61	0.07	0.60	0.08	0.77	0.36	0.76	0.34	0.69	0.20	0.69	0.19	0.66	0.14	0.67	0.17
**Fairly high(2)**	0.63	0.16	0.57	0.09	0.94	0.87	0.87	0.71	0.85	0.62	0.81	0.54	0.74	0.36	0.73	0.35
**Very high(3)**	0.40	0.05	0.35	0.03	0.57	0.23	0.63	0.34	0.64	0.35	0.74	0.53	0.53	0.18	0.62	0.31

a: crude model,

b: adjusted for age, sex and BMI .

**Table 4.** Linear regression predicting blood pressure and heart rate parameters by sleep data

	**Systolic**				**Diastolic**				**MHR**			

	β^a^	p–Value^a^	β^b^	p-Value^b^	β^a^	p–Value^a^	β^b^	p-Value^b^	β^a^	p–Value^a^	β^b^	p-Value^b^
**PSQI (continue)**	0.63	0.05	0.23	0.45	0.51	0.03	0.10	0.67	0.65	0.005	0.33	0.15
**PSQI**												
**GOOD(N= 80)<5 0 Reference**												
**POOR(N=104)>5 1**	3.43	0.15	2.15	0.32	3.61	0.04	1.96	0.25	3.52	0.04	1.74	0.28
**Subjective sleep quality**												
**New-E9**												
**Very good (0) Reference**												
**Fairly good (1)**	1.44	0.57	3.59	0.12	1.22	0.53	2.12	0.24	3.6	0.04	2.53	0.13
**Fairly bad (2)**	4.84	0.13	4.12	0.15	2.73	0.26	1.69	0.46	7.06	0.001	4.62	0.03
**Very bad (3)**	−0.82	0.86	−3.32	0.41	−1.27	0.72	−3.37	0.30	4.86	0.12	3.33	0.26
**Sleep latency**												
**Very low (0) Reference**												
**Fairly low (1)**	−0.15	0.96	0.82	0.73	−2.69	0.17	−1.73	0.34	3.84	0.03	2.98	0.08
**Fairly high (2)**	−0.19	0.70	−1.19	0.66	−1.26	0.58	−0.64	0.76	5.4	0.009	4.37	0.025
**Very high (3)**	1.38	0.71	−0.14	0.96	−0.52	0.85	−1.44	0.57	6.97	0.005	4.74	0.045
**Sleep duration**												
**>=7 h (0) Reference**												
**6–7 h (1)**	−1.64	0.62	−0.69	0.83	0.99	0.70	0.88	0.72	0.65	0.79	−1.24	0.59
**5–6 h (2)**	3.96	0.14	2.97	0.23	3.05	0.14	1.57	0.43	1.001	0.61	−0.001	1.00
**<5 h (3)**	7.3	0.02	3.88	0.19	4.18	0.08	0.65	0.78	0.05	0.98	−2.32	0.29
**habitual sleep efficiency**												
**>85% (0) Reference**												
**75–84% (1)**	3.04	0.32	1.12	0.68	−0.02	0.99	−2.05	0.35	−4.05	0.07	−4.09	0.052
**65–74% (2)**	1.83	0.61	1.72	0.59	0.44	0.87	0.12	0.96	−0.39	0.88	−1.69	0.48
**<65% (3)**	5.48	0.08	1.97	0.48	5.00	0.03	1.82	0.41	3.3	0.14	1.11	0.60
**Sleep disturbances**												
**Very Low (0)**												
**Fairly Low (1)**	6.13	0.17	7.09	0.09	7.44	0.03	6.65	0.04	0.69	0.83	−0.96	0.75
**Fairly High (2)**	5.12	0.29	5.16	0.26	6.48	0.08	4.96	0.16	4.15	0.22	0.75	0.82
**Very High (3)**	15.2	0.02	4.19	0.44	10.7	0.03	1.47	0.77	6.7	0.15	0.02	0.99
**Use of sleeping medication**												
**Not during the past month (0)**												
**Reference**												
**Less than once a week (1)**	2.8	0.63	0.40	0.94	6.22	0.16	4.65	0.25	15.4	<0.001	16.4	<0.001
**Once or twice a week (2)**	13.3	0.07	6.92	0.29	12.74	0.02	8.77	0.09	3.7	0.46	1.22	0.79
**Three or more times a week (3)**	−2.97	0.48	−3.61	0.34	0.13	0.97	−0.31	0.92	3.84	0.18	3.01	0.26
**Daytime dysfunction**												
**Very low (0) Reference**												
**Fairly low (1)**	−1.64	0.51	0.31	0.89	−0.54	0.77	0.09	0.96	3.19	0.06	1.37	0.39
**Fairly high (2)**	0.25	0.93	1.81	0.49	1.75	0.43	2.47	0.23	3.1	0.13	0.64	0.73
**Very high (3)**	6.71	0.11	1.86	0.63	0.02	0.99	−4.15	0.17	6.8	0.02	4.27	0.12

## DISCUSSION

According to our study, poor sleep quality is adversely associated with HRV, HR and BP. The association of sleep and HRV has been a matter of debate. It is known that heart rate decreases during sleep and generally follows the circadian curve of body temperature. However, within this overall pattern, heart rate is also related to sleep stage, awakenings, and body movements. Sleep stages alternate throughout a normal sleep period and correlate with changes in HRV. During non-rapid eye movement (NREM) sleep stage, the cardiovascular system is stable and parasympathetic cardiac modulation is stronger. During rapid eye movement (REM) sleep stage, the cardiovascular system is unstable and greatly influenced by surges in sympathetic activity (8, 14–16). In an old study conducted by Zemaityte et al. in 1984, HRV and respiratory changes in limited number of healthy subjects during their sleep were assessed. They found that during REM stage of sleep HR would increase, but generally HRV was dependent on basal autonomic activity of each patient (17). Crasset et al. evaluated the aging effect on HRV during sleep. They reported that during Non-REM sleep, old people had shorter RR intervals and lower HRV compared to young subjects (18).

Stress is a factor that has been considered as a risk of sleep disturbances and thus may affect persons' autonomic function during sleeping and influence HRV during sleeping. Previous studies have presented that acute stress can cause autonomic dysfunction during sleep. In fact healthy cases who were facing stressful conditions were struggling with decreased parasympathetic modulation during both REM and non-REM phases of their sleep. In addition they were struggling with increased sympathovagal balance disturbances during their non-REM phase of sleep. In agreement with our study, in one published study based on polysomnography HRV was increased in association with poor sleep maintenance and sleep disturbances (19). In another study conducted by Davidson et al. (20), sleep disturbances of residents of areas with presentation of chronic stress was assessed and compared with normal population as controls. They found modest association of sleep disturbances with stressful life and concluded that basal stress could be considered as a possible factor, but may not play the main role in sleep disturbances (20). There are large number of further studies in which they have concluded that stress and negative thoughts can affect sleep onset and also quality (21, 22). We did not measure stress level in our study, but our findings were in complete agreement with these previous studies.

Sleep problems could result in autonomic imbalance as well, but presentations of previous studies are controversial (6). In a study conducted by Holmes et al. showed that an acute deprivation of sleeping was accompanied with decreased cardiac sympathetic activity while parasympathetic activity did not change (23). These findings were inconsistent with the study conducted by Xu Zhong et al. that found increased sympathetic and reduced parasympathetic activity following 36 hours of sleep deprivation (24). A more recent investigation of the effects of sleep deprivation on autonomic regulation found that sleep deprivation predicted an increase in vagal tone (25). Our finding in this study shows both sympathetic and parasympathetic activity changes with sleep duration.

Higher HR and lower HRV during sleep are considered as independent risk factors of cardiovascular diseases. In fact cardiac autonomic dysregulation is considered as risk factor of cardiovascular diseases during awakenings and also sleeping. This is mentioned in study of Brosschot et al. that low HRV is accompanied with higher risks of morbidity and mortality (21). Another study assessed sleep disturbances among males with metabolic syndrome and sleep apnea; they presented that diurnal pattern of sympathovagal imbalance among these cases-due to their nocturnal sleep disturbances- is accompanied with parasympathetic loss during sleep and decreased HRV. In fact, they concluded that reduced HRV is independent of underlying obstructive sleep apnea and accompanied with higher morbidity and mortality in this group (26).

The association of ANS and BP has been known for a long time. SNS raises the BP; whereas PNS lowers it. Studying both HR and BP fluctuations is known to be a method for investigating the ANS (27). Moreover, the association of sleep and BP has been studied before. Sleep in healthy humans is associated with a decrease in HR, BP, and sympathetic activity; whereas it seems that it is associated with increased parasympathetic activity (28). Experimental data suggest that reducing sleep length may result in adverse responses, such as an increase in HR (28). Sleep problems could result in autonomic imbalance as well, for instance by increasing the dominance of sympathetic activity over parasympathetic modulation (6). Previously it has been presented that sleep deprivation can cause hypertension in normal population. The study of Robillard et al. found that sleep duration of seven-hours-aday and less is accompanied with hypertension. They stated that in case of less than six-hours-a-day sleeping, this association would be more steeply. This study confirms the influence of sleep duration on BP (29). Various mechanisms such as the association of sleep duration and hypothalamic–pituitary– adrenal axis and also renin–angiotensin system have been suggested as the causal link between sleep duration and hypertension (28). Kuciene and Dulskiene (30) found that short sleep duration was associated with higher BP, while other studies reported a positive (31) or no association (32) between sleep duration and BP.

Previous investigations have presented that cases with sleep disorders are prone to hypertension. Studies on cases with breathing disorders have mentioned this association overtly, as this hypertension may have occurred due to their underlying breathing disorder regardless of their sleep disorders (29). Other studies conducted on normal populations have presented that cases complaining from low quality of their sleep are highly prone to hypertension (33). Moreover, Bruno et al. presented that cases with hypertension who were resenting from low quality sleep were significantly more prone to poor BP treatment response (34).

Our study had some limitations including that we did not exclude obstructive sleep apnea as a confounding factor. The cross-sectional nature of the study is another limitation. Repeated recordings of HRV in different times of the day would be analytically more valuable. The lack of a control group was also a flaw in this study. Its strength is the novelty of the variables studied.

## CONCLUSION

Our finding showed that poor sleep quality is adversely associated with HRV, HR and BP. This finding should be considered in clinical and preventive recommendations.

### Ethics approval and consent to participate

This study was ethically approved by “the Ethics Committee of Isfahan University of Medical Sciences”. Informed written consent was obtained from all of the participants.
